# The use of graphics in decision making

**DOI:** 10.1590/0100-6991e-20213000

**Published:** 2021-04-24

**Authors:** ÁLIDA ROSÁRIA SILVA FERREIRA

**Affiliations:** 1 - Universidade Federal de Minas Gerais (UFMG) - Belo Horizonte - MG - Brasil

The presentation of data through graphics is a strategy that can cause problems, impacting the interpretation of the study results if not used with caution.

The first important aspect is understanding the graphic itself does not allow statistical conclusions to be reached. Differences can only be identified when the correct analysis is carried out, and this is done through the p-value, which will confirm whether the groups’ behavior is similar.

Expressions such as “the graphic shows a trend” are erroneous since the statistical analysis indicates the occurrence probabilities that will reflect not into a certainty but a trend.

As the values of the mean, median, and other dispersion measures cannot be parameters to evaluate the differences, the graphics are also inadequate. In case the statistical analysis identifies such differences, the graphic will have a confirmation role, not a conclusion one.

Another important point regarding graphics is related to its interpretation. A graphic must be self-explanatory; that is, it should depend as little as possible on the supporting text. If that is not possible, this may not be the best strategy to present the data.

Another suggestion is to pay attention to the graphic scales; once next to each other, they must respect the same pattern of scales to not lead to false conclusions. Do not trust the programs that generate automatic graphics and make this adjustment manually to make the reading as straightforward as possible.

Finally, if the data are already displayed in a table, do not repeat them in a graphic. The objective of both is the same, and doing so will lead to duplicate information that will use important space for the article’s discussions.
